# Genetic diversity and population structure of *Urochloa* grass accessions from Tanzania using simple sequence repeat (SSR) markers

**DOI:** 10.1007/s40415-018-0482-8

**Published:** 2018-07-24

**Authors:** S. O. Kuwi, M. Kyalo, C. K. Mutai, A. Mwilawa, J. Hanson, A. Djikeng, S. R. Ghimire

**Affiliations:** 1 The Bioscience eastern and central Africa - International Livestock Research Institute Hub, Nairobi, Kenya; 2 Tanzania Livestock Research Institute, Dodoma, Tanzania; 3 International Livestock Research Institute, Addis Ababa, Ethiopia

**Keywords:** Apomixis, *Brachiaria*, Carbon sequestration, Polyploid, Principal coordinate analysis, Private alleles

## Abstract

*Urochloa* (syn.—*Brachiaria* s.s.) is one of the most important tropical forages that transformed livestock industries in Australia and South America. Farmers in Africa are increasingly interested in growing *Urochloa* to support the burgeoning livestock business, but the lack of cultivars adapted to African environments has been a major challenge. Therefore, this study examines genetic diversity of Tanzanian *Urochloa* accessions to provide essential information for establishing a *Urochloa* breeding program in Africa. A total of 36 historical *Urochloa* accessions initially collected from Tanzania in 1985 were analyzed for genetic variation using 24 SSR markers along with six South American commercial cultivars. These markers detected 407 alleles in the 36 Tanzania accessions and 6 commercial cultivars. Markers were highly informative with an average polymorphic information content of 0.79. The analysis of molecular variance revealed high genetic variation within individual accessions in a species (92%), fixation index of 0.05 and gene flow estimate of 4.77 showed a low genetic differentiation and a high level of gene flow among populations. An unweighted neighbor-joining tree grouped the 36 accessions and six commercial cultivars into three main clusters. The clustering of test accessions did not follow geographical origin. Similarly, population structure analysis grouped the 42 tested genotypes into three major gene pools. The results showed the *Urochloa brizantha* (A. Rich.) Stapf population has the highest genetic diversity (*I* = 0.94) with high utility in the *Urochloa* breeding and conservation program. As the *Urochloa* accessions analyzed in this study represented only 3 of 31 regions of Tanzania, further collection and characterization of materials from wider geographical areas are necessary to comprehend the whole *Urochloa* diversity in Tanzania.

## Introduction

*Urochloa* (syn.—*Brachiaria* s.s.) that consists of about 100 species is among the most widely cultivated tropical forage grass in South America, Australia and East Asia and has been recognized for high yield, nutritional content and wider adaptability to diverse ecological niches (Miles et al. 1996). *Urochloa* is a tropical warm season forage native to Africa and was first introduced to Australia in about 1800 (Barnard 1969) and subsequently into tropical South America during the mid-nineteenth century (Parsons 1972). *Urochloa* is resistant to drought, insect pests and diseases and competes effectively with other plant species and quickly covers the ground (Stomayor-Rios et al. 1960). *Urochloa* produces a yearly dry forage yield of 5–36 t/ha depending on soil fertility, soil moisture content and fertilizer application (Bogdan 1977). The forage is palatable and highly nutritious contributing to a significant increase in livestock milk and meat production. Moreover, *Urochloa* sequesters carbon, enhances N use efficiency through a biological nitrification inhibition process and subsequently reduces greenhouse gas emission and groundwater pollution (Subbarao et al. 2009; Danilo et al. 2014; Arango et al. 2014).

Low livestock productivity is a common feature across sub-Saharan Africa (SSA) contributed largely by shortage of quality feed particularly during the dry seasons. Though not a tradition, farmers have started growing improved forages to support the emerging livestock sector in the region. Recently, *Urochloa* has emerged as one of the important forage options among smallholder farmers of Africa (Ghimire et al. 2015). However, the wider adoption of *Urochloa* grass in Africa is constrained by unavailability of seeds, lack of improved agronomic practices and nonexistence of a variety suitable for wide-ranging environments. The varieties currently introduced to Africa were developed in Australia and tropical America from the African germplasm. The commercial cultivation of these varieties developed elsewhere can lead to an elevated risk of pests and diseases, and of poor adaptation to other biotic and abiotic stresses. Therefore, the need for Africa-based *Urochloa* breeding program accommodating natural genetic diversity in the region has been recently realized with the aim to develop varieties suitable to different production environments.

The characterization of genetic diversity of a population is necessary for better use of genetic resources in breeding and biodiversity conservation programs. Therefore, knowledge of genetic diversity of the available germplasm is essential in selecting materials for cultivation or parents for cultivar development. The genetic diversity can be assessed using different tools including DNA markers (Kapila et al. 2008). Molecular markers are valuable tools for characterization and evaluation of genetic diversity within and between species and populations. Different molecular markers such as random amplified polymorphic DNA (RAPD), inter-simple sequence repeats (ISSR), simple sequence repeats (SSR) and amplified fragment length polymorphism (AFLP) have been used to assess the genetic diversity in plant species (Balasaravanan et al. 2003; Khan et al. 2005; Terzopoulos et al. 2005) of which the simple sequence repeats (SSR) are preferred due to ease of application, high reproducibility, rapid analysis, low cost, easy scoring patterns and higher allelic diversity (Chen et al. 1997). The SSR markers are codominant markers that can detect both homozygote and heterozygote individuals and are distributed throughout the genome (McCouch et al. 1997). Knowing the degree of genetic differences among *Urochloa* genotypes is useful to organize a working collection and to select genotypes for crossing and conservation (Mendes-Bonato et al. 2006). Despite the importance of *Urochloa*, limited information is available on biology and genetic diversity of the genus, which has severely constrained the breeding and conservation efforts. Therefore, this study was conducted to assess the genetic diversity and population structure of Tanzanian *Urochloa* accessions from the historical collection maintained at the Field Genebank of the International Livestock Research Institute (ILRI), Ethiopia. The result of this study would be highly useful in a *Urochloa* improvement and conservation program.

## Materials and methods

**Fig. 1:**
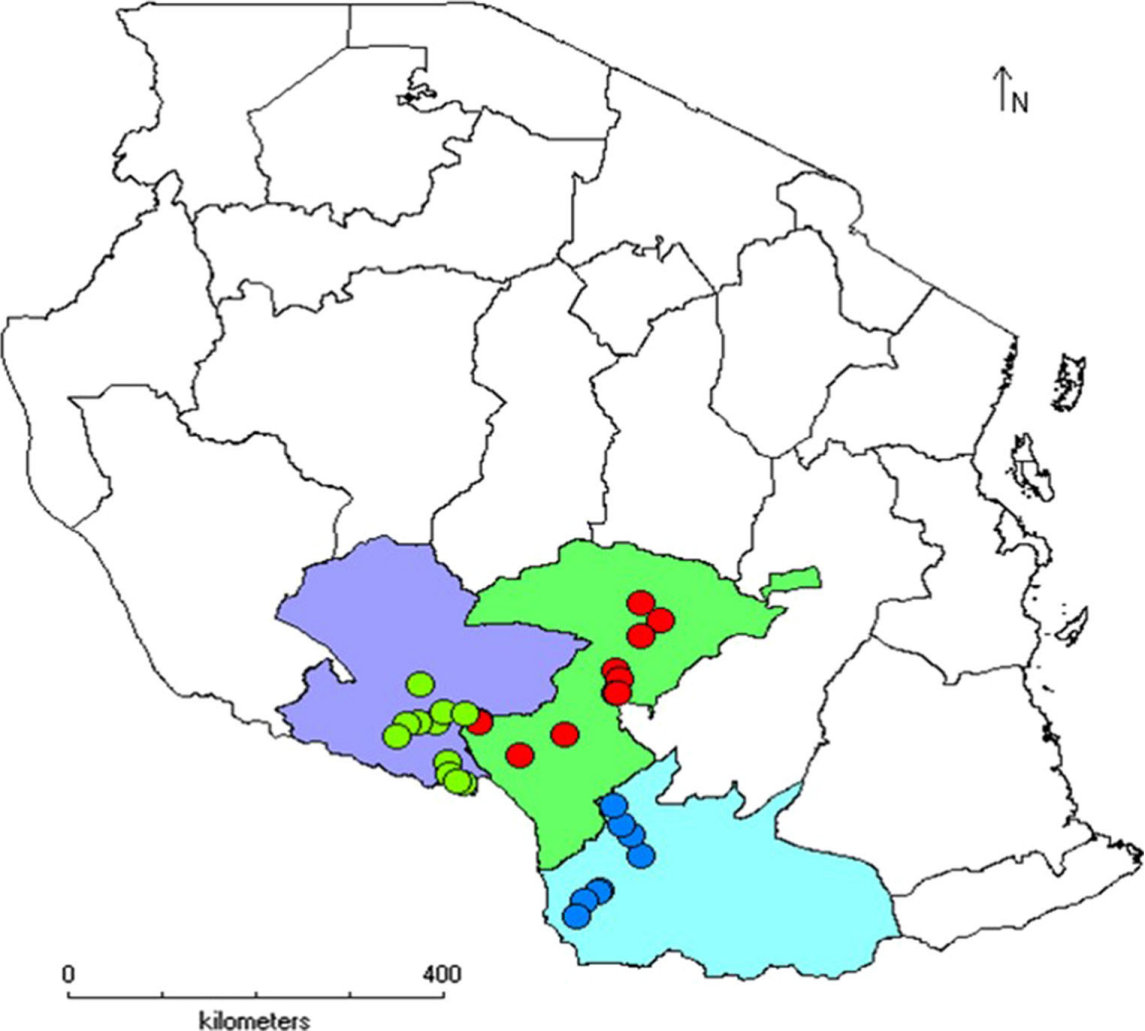
Map of Tanzania showing the origin of *Urochloa* accessions. Purple, blue and green colors in map represent Mbeya, Iringa and Ruvuma regions, respectively. (Color figure online)

**Source of plant materials** – A total of 36 *Urochloa* accessions originally collected from Tanzania and six commercial cultivars (Basilisk, Humidicola, Llanero, MG4, Mulato II and Piata) were included in this study (Table 1). The Genbank accessions were collected from natural populations from the Iringa, Mbeya and Ruvuma regions of Tanzania (Fig. 1) during 1985 and since then maintained in ILRI’s Forage Field Genebank at Zwai, Ethiopia. Fresh young leaf samples were collected, dried in silica gel and transported to the Biosciences eastern and central Africa—International Livestock Research Institute (BecA-ILRI) Hub, Nairobi, Kenya, for subsequent analysis. Leaf samples of six commercial cultivars were collected from pasture evaluation plots at ILRI Headquarters, Nairobi, Kenya.

**Table 1:** Details of *Urochloa* accessions and commercial cultivars used in the study

S. no	Accession	Other ID #	Species	Variety	Origin	Region	Latitude	Longitude	Collection year
1	ILCA-814	CIAT 26386	*U. brizantha*	NA	Tanzania	Iringa	– 8.89	33.98	1985
2	ILCA-726	CIAT 26370	*U. brizantha*	NA	Tanzania	Iringa	– 7.9501	35.56	1985
3	ILCA-731	CIAT 26371	*U. brizantha*	NA	Tanzania	Iringa	– 8.3298	35.3104	1985
4	ILCA-869	CIAT 26397	*U. brizantha*	NA	Tanzania	Mbeya	– 8.5	33.4	1985
5	ILCA-717	CIAT 26407	*U. humidicola*	NA	Tanzania	Iringa	– 7.78	35.75	1985
6	ILCA-10871	–	*U. decumbens*	Basilisk	Uganda	NA	NA	NA	NA
7	ILCA-12470	–	*U. humidicola*	Llanero	Zambia	NA	NA	NA	NA
8	ILCA-828	CIAT 26389	*U. brizantha*	NA	Tanzania	Mbeya	– 8.92	33.39	1985
9	ILCA-821	CIAT 26388	*U. brizantha*	NA	Tanzania	Mbeya	– 8.82	33.84	1985
10	ILCA-849	CIAT 26393	*U. brizantha*	NA	Tanzania	Mbeya	– 9.35	33.67	1985
11	–	CIAT 16125	*U. brizantha*	Piata	–	NA	NA	NA	NA
12	ILCA-758	–	*U. jubata*	NA	Tanzania	Ruvuma	– 10.77	35.13	1985
13	ILCA-767	CIAT 26380	*U. brizantha*	NA	Tanzania	Ruvuma	– 10.3718	35.5573	1985
14	ILCA-781	CIAT 26381	*U. brizantha*	NA	Tanzania	Ruvuma	– 10.0266	35.3737	1985
15	ILCA-785	CIAT 26384	*U. brizantha*	NA	Tanzania	Iringa	– 9.28	34.38	1985
16	ILCA-732	CIAT 26434	*U. ruziziensis*	NA	Tanzania	Iringa	– 8.3298	35.3104	1985
17	ILCA-829	CIAT 26423	*U. humidicola*	NA	Tanzania	Mbeya	– 8.93	33.27	1985
18	ILCA-728	CIAT 26411	*U. humidicola*	NA	Tanzania	Iringa	– 7.9501	35.56	1985
19	ILCA-727	CIAT 26438	*U. bovonei*	NA	Tanzania	Iringa	– 7.9501	35.56	1985
20	ILCA-735	CIAT 26414	*U. humidicola*	NA	Tanzania	Iringa	– 8.5853	35.3122	1985
21	ILCA-822	CIAT 26422	*U. humidicola*	NA	Tanzania	Mbeya	– 8.82	33.84	1985
22	ILCA-853	CIAT 26427	*U. humidicola*	NA	Tanzania	Mbeya	– 9.48	33.7	1985
23	ILCA-864	CIAT 26430	*U. humidicola*	NA	Tanzania	Mbeya	– 9.55	33.76	1985
24	ILCA-832	CIAT 26424	*U. humidicola*	NA	Tanzania	Mbeya	– 9.0549	33.1715	1985
25	ILCA-857	CIAT 26428	*U. humidicola*	NA	Tanzania	Mbeya	– 9.57	33.83	1985
26	–	CIAT 36087	*U. hybrid*	Mulato-II	Colombia	NA	NA	NA	NA
27	ILCA-810	CIAT 26385	*U. brizantha*	NA	Tanzania	Mbeya	– 8.91	33.56	1985
28	ILCA-756	CIAT 26404	*U. brizantha*	NA	Tanzania	Ruvuma	– 10.76	35.16	1985
29	ILCA-769	CIAT 26439	*U. bovonei*	NA	Tanzania	Ruvuma	– 10.1543	35.4718	1985
30	ILCA-815	CIAT 26420	*U. humidicola*	NA	Tanzania	Iringa	– 8.89	33.98	1985
31	ILCA-734	CIAT 26413	*U. humidicola*	NA	Tanzania	Iringa	– 8.4303	35.3511	1985
32	ILCA-819	CIAT 26421	*U. humidicola*	NA	Tanzania	Iringa	– 8.91	33.98	1985
33	ILCA-782	CIAT 26382	*U. brizantha*	NA	Tanzania	Ruvuma	– 9.82	35.3	1985
34	ILCA-760	CIAT 26378	*U. brizantha*	NA	Tanzania	Ruvuma	– 10.88	35.01	1985
35	ILCA-744	CIAT 26416	*U. humidicola*	NA	Tanzania	Iringa	– 9.0392	34.8211	1985
36	ILCA-736	CIAT 26415	*U. humidicola*	NA	Tanzania	Iringa	– 8.5798	35.324	1985
37	ILCA-863	CIAT 26396	*U. brizantha*	NA	Tanzania	Mbeya	– 9.55	33.76	1985
38	ILCA-718	CIAT 26408	*U. humidicola*	NA	Tanzania	Iringa	– 7.6006	35.5495	1985
39	ILCA-761	CIAT 26379	*U. brizantha*	NA	Tanzania	Ruvuma	– 11.04	34.92	1985
40	–	CIAT 26646	*U. brizantha*	MG4	Trinidad	NA	NA	NA	NA
41	ILCA-812	CIAT 26405	*U. brizantha*	NA	Tanzania	Mbeya	– 8.8	33.64	1985
42	–	CIAT 679	*U. humidicola*	Humidicola	South Africa	NA	NA	NA	NA

*NA* not available

**Genomic DNA extraction** – Genomic DNA was extracted from dried leaves using Zymo extraction kit (Zymo Research, USA) according to manufacturer’s instructions. The quality, quantity and integrity of DNA were estimated using the NanoDrop 1000 Spectrophotometer (Thermo Fisher Scientific, Waltham, MA) and visualized in 1% agarose gel (w/v) stained with 0.25X GelRed under ultraviolet light (UVP BioImaging Systems, Upland, CA). The DNA was adjusted to the final concentration of 20 ng/μl and stored at – 20 °C until further use.

**PCR amplification and capillary electrophoresis** – A total of 24 SSR markers initially developed for U. ruziziensis Germain & Evrard with the proven transferability to other *Urochloa* species were used in the study (Silva et al. 2013; Table 2). Primers were optimized for appropriate annealing temperature using gradient PCR. Thereafter, multiplex PCR was used to amplify genomic DNA using AccuPower® PCR PreMix without dye (Bioneer, Republic of Korea). PCR amplification was performed in a final reaction volume of 10 μl containing 40 ng genomic DNA, 0.09 μM of each forward and reverse primer (labeled with different fluorescent dyes: 6-FAM, VIC, NED and PET), 0.5 μM MgCl_2_ and 7.2 μl sterile water. The PCR amplifications were performed in a GeneAmp PCR System 9700 thermocycler (Applied Biosystems, Foster City, CA) using the following PCR cycling conditions: initial denaturation at 95 °C for 3 min, followed by 35 cycles of 94 °C for 30 s, annealing at 58/59 °C for 1 min, extension at 72 °C for 2 min and final extension at 72 °C for 20 min and hold at 15 °C. The amplicons were separated in 2% agarose gel stained with 0.25 × GelRed and run for 45 min at 100 V. A cocktail (LH) of 15 μl GeneScan^TM^500LIZ size standard (Applied Biosystems, USA) and 1 ml Hi-Di-formamide was prepared for capillary electrophoresis. Multiplexed PCR product (1.5 μl) was mixed with 9 μl of LH, denatured at 95 °C for 3 min and snap-chilled on ice for 5 min. The samples were then subjected to capillary electrophoresis at the Segolip Unit of BecA-ILRI HuU.

**Table 2:** SSR markers used for the genetic diversity study of Tanzania *Urochloa* accessions and commercial varieties (adapted from Silva et al. 2013)

Marker	Forward primer sequence	Reverse primer sequence	Annealing temperature (°C)	Expected product size range (bp)	Repeat motif
Br0012	ACTCAAACAATCTCCAACACG	CCCCACAAATGGTGAATGTAAC	59	144–196	(AT)_8_
Br0028	CATGGACAAGGAGAAGATTGA	TGGGAGTTAACATTAGTGTTTTT	58	111–197	(TA)_8_
Br0029	TTTGTGCCAAAGTCCAAATAG	TATTCCAGCTTCTTCTGCCTA	59	132–178	(AG)_14_
Br0031	CCCCCATTTAACACCATAGTT	GCTCAAAATGCAATGTACGTG	59	139–179	(AT)_9_
Br0067	TTAGATTCCTCAGGACATTGG	TCCTATATGCCGTCGTACTCA	59	130–171	(AT)_9_
Br0076	CCTAGAATGCGGAAGTAGTGA	TTACGTGTTCCTCGACTCAAC	59	120–262	(AT)_7_
Br0087	TTCCCCCACTACTCATCTCA	AACAGCACACCGTAGCAAGT	58	229–261	(GA)_9_
Br0092	TTGATCAGTGGGAGGTAGGA	TGAAACTTGTCCCTTTTTCG	59	200–295	(AT)_6_
Br0100	CCATCTGCAATTATTCAGGAAA	GTTCTTGGTGCTTGACCATT	58	229–286	(AT)_11_
Br0115	AATTCATGATCGGAGCACAT	TGAACAATGGCTTTGAATGA	59	231–315	(AT)_6_
Br0117	AGCTAAGGGGCTACTGTTGG	CGCGATCTCCAAAATGTAAT	59	233–345	(TA)_5_
Br0118	AGGAGGTCCAAATCACCAAT	CGTCAGCAATTCGTACCAC	59	237–321	(CT)_11_
Br0156	CATTGCTCCTCTCGCACTAT	CTGCAGTTAGCAGGTTGGTT	58	223–279	(CA)_6_
Br0130	TCCTTTCATGAACCCCTGTA	CATCGCACGCTTATATGACA	58	199–299	(CT)_14_
Br0149	GCAAGACCGCTGTTAGAGAA	CTAACATGGACACCGCTCTT	58	231–299	(AT)_11_
Br0152	ATGCTGCACTTACTGGTTCA	GGCTATCAATTCGAAGACCA	58	233–301	(TC)_11_
Br0214	GCCATGATGTTTCATTGGTT	TTTTGCACCTTTCATTGCTT	59	231–286	(AC)_7_
Br0203	CGCTTGAGAAGCTAGCAAGT	TAGCCTTTTGCATGGGTTAG	58	208–310	(GA)_8_
Br0212	ACTCATTTTCACACGCACAA	CGAAGAATTGCAGCAGAAGT	58	248–330	(CA)_5_
Br0213	TGAAGCCCTTTCTAAATGATG	GAACTAGGAAGCCATGGACA	58	212–337	(CA)_7_
Br0122	TCTGGTGTCTCTTTGCTCCT	TCCATGGTACCTGAATGACA	58	241–358	(AT)_8_
Br0235	CACACTCACACACGGAGAGA	CATCCAGAGCCTGATGAAGT	59	239–330	(TC)_9_
Br3002	GCTGGAATCAGAATCGATGA	GAACTGCAGTGGCTGATCTT	59	143–187	(AAT)_7_
Br3009	AGACTCTGTGCGGGAAATTA	ACTTCGCTTGTCCTACTTGG	58	116–199	(AAT)_10_

**Data analysis** – Forty-two *Urochloa* genotypes consisting five species: *U. bovonei* (Chiov.) Robyns, *U. brizantha* (A. Rich.) Stapf, *U. jubata* (Fig. & De Not.) Stapf, *U. humidicola* (Rendle) Schweick, *U. ruziziensis* Germain & Evrard and six *Urochloa* cultivars were grouped into six populations for the genetic diversity study. The descriptive statistics for SSR markers were computed with PowerMarker v.3.25 software (http://www.powermarker.net). The population diversity description, principal coordinate analysis (PCoA) and analysis of molecular variance (AMOVA) were performed using GenAlEx v6.41 (Peakall and Smouse 2006). The neighbor-joining method (NJ) was used to generate the dendrogram using Darwin v.6.0.010 (Perrier and Jacquemoud-Collet 2006). One thousand bootstrap replicates were used to determine branch support in the consensus tree. Structure v.2.3.4 (Pritchard et al. 2000) was used to infer the population structure and ancestry of samples based on Bayesian statistics. The parameter set for this analysis used the admixture model, and batch runs with correlated and independent allele frequencies among inferred populations were tested with burn-in and run length of 50,000 and 100,000, respectively. All other parameters were set to default values. A batch job with values of *K* ranging from 1 to 10 was set up, with ten independent runs for each successive *K*. This procedure clusters individuals into populations and estimates the proportion of membership in each population for every individual. The *K* value was determined by the log probability of data [(Ln P(D)] based on the rate of change in Ln P(D) between successive *K*. The optimum *K* value was predicted following the simulation method (Evanno et al. 2005) using the web-based software Structure Harvester v.0.6.92 (Earl and Von Holdt 2012).

## Results

**SSR polymorphism and genetic diversity** – A total of 407 alleles ranging in size from 111 to 345 bp were detected (Tables 2, 3). The number of alleles scored per locus varied from 5 (Br0067) to 40 (Br0028) with an average of 16.96 alleles across all loci. The PIC value varied from 0.64 (Br0213) to 0.95 (Br0235) with an average of 0.79 per locus (Table 3).

**Table 3:** Diversity indices for 24 microsatellite markers

SSR locus	*N_A_*	*N_DA_*	*I*	*H_O_*	*H_E_*	PIC
Br0012	9	9	0.86	0.50	0.35	0.85
Br0029	11	10	0.73	0.42	0.26	0.71
Br0031	9	9	0.67	0.50	0.36	0.66
Br0067	5	5	0.84	0.17	0.11	0.82
Br0076	9	9	0.81	0.50	0.33	0.80
Br0087	22	16	0.75	0.43	0.31	0.74
Br0092	7	7	0.76	0.50	0.30	0.74
Br0115	16	12	0.95	0.66	0.44	0.95
Br0117	8	8	0.74	0.50	0.31	0.73
Br0118	12	11	0.70	0.42	0.24	0.68
Br0212	17	13	0.95	0.57	0.44	0.95
Br0214	16	12	0.93	0.88	0.66	0.92
Br0235	31	23	0.95	0.71	0.55	0.95
Br3002	11	9	0.87	0.63	0.46	0.86
Br0028	40	19	0.79	0.65	0.48	0.78
Br0100	13	13	0.73	0.63	0.44	0.72
Br0122	10	9	0.78	0.42	0.26	0.76
Br0130	18	11	0.76	0.67	0.47	0.75
Br0149	13	12	0.67	0.50	0.32	0.66
Br0152	29	19	0.73	0.69	0.43	0.73
Br0156	38	22	0.85	0.74	0.55	0.85
Br0203	22	15	0.80	0.56	0.36	0.79
Br0213	6	6	0.66	0.50	0.32	0.64
Br3009	35	23	0.81	0.70	0.49	0.80
Mean	16.96 ± 10.43	12.74 ± 5.32	0.80 ± 0.09	0.56 ± 0.15	0.38 ± 0.12	0.79 ± 0.09

*N_A_* number of alleles, *N_DA_* number of different alleles, *I* Shannon index, *H_O_* observed heterozygosity, *H_E_* expected heterozygosity

**Population genetic diversity** – The genetic diversity indices for *Urochloa* populations are summarized in Table 4. The average number of effective alleles (*N_E_*), number of private alleles (*N_P_*) and percentage of polymorphic loci (%PL) across all loci ranged from 0.34–2.74, 0.08–1.53 and 17.19–68.75%, respectively, in the studied populations. The observed heterozygosity (*H_O_*) was in the range of 0.17–0.69, with a mean of 0.49. The high-level diversity was observed in *U. brizantha* population (*I* = 0.94) and a low-level diversity in *U. bovonei* population (*I* = 0.12). The observed heterozygosity was higher than expected for all populations.

**Table 4:** Summary of population genetic diversity indices averaged over 24 SSR markers

Population	*N*	*N_A_*	*N_E_*	*N_P_*	I	*H_O_*	*H_E_*	%PL
*U. brizantha*	17	3.50	2.74	1.53	0.94	0.69	0.47	68.75
*U. humidicola*	15	2.70	2.37	1.03	0.77	0.58	0.40	57.81
*U. bovonei*	2	0.94	0.69	0.22	0.25	0.31	0.17	31.25
*U. ruziziensis*	1	0.34	0.34	0.08	0.12	0.17	0.09	17.19
*U. jubata*	1	0.59	0.59	0.28	0.21	0.30	0.15	29.67
*Urochloa Cultivars*	6	1.61	1.48	0.66	0.52	0.46	0.30	46.88
Mean	8.4	1.89	1.64	0.76	0.56	0.49	0.31	41.93
SE (±)	0.13	0.12	0.10	0.12	0.03	0.03	0.02	7.90

*N* number of accessions, *N_A_* number of alleles, *N_E_* number of effectives alleles, *I* information index, *H_O_* observed heterozygosity, *H_E_* expected heterozygosity, *N_P_* number of private alleles, *%PL* percentage of polymorphic loci

**Genetic distance** – The pairwise genetic distance and population matrix of Nei unbiased genetic identity were presented in Table 5. Among four populations analyzed (excluding *U. ruziziensis* and *U. jubata*), *U. bovonei* and commercial cultivar populations were distantly related (3.186), whereas *U. brizantha* and *U. humidicola* populations were the most closely related (1.639). Similarly, genetic identity was the highest between *U. brizantha* and *U. humidicola* populations (0.194) and the lowest between *U. bovonei* and commercial cultivar populations (0.041).

**Table 5:** Pair-wise genetic distance based on shared allele (below diagonal) and genetic identity among *Urochloa* populations (above diagonal)

Population	*U. brizantha*	*U. humidicola*	*U. bovonei*	Cultivars
*U. brizantha*	–	0.194	0.048	0.097
*U. humidicola*	1.639	–	0.055	0.067
*U. bovonei*	3.044	2.893	–	0.041
Cultivars	2.333	2.709	3.186	–

**Analysis of molecular variance** – Analysis of molecular variance (AMOVA) of 42 *Urochloa* genotypes showed that only 3% of the total variation in the population was due to differences among individual accessions. Differences within individual accessions in a population contributed 94% of total variation, and 5% was due to the differences among the *Urochloa* populations (Table 6). There was a low genetic differentiation in the total populations (*F*_ST-_ = 0.05) as evidenced by high level of gene flow estimate (Nm = 4.77).

**Table 6:** Analysis of molecular variance (AMOVA) of populations of *Urochloa* accessions and cultivars based on 24 SSR loci

Source	Degree of freedom	Sum of squared	Mean of squared	Estimated variance	Variation (%)	*P* values
Among populations	4	96.841	24.210	0.712	5	0.001
Among individuals	37	517.754	13.993	0.407	3	0.084
Within individuals	42	553.500	13.179	13.179	92	0.001
Total	83	1168.095		14.298	100	
*F_ST_* = 0.05; Nm = 4.77						

*F*_ST_ Fixation index, *Nm* Number of migration per generation

**Fig. 2:**
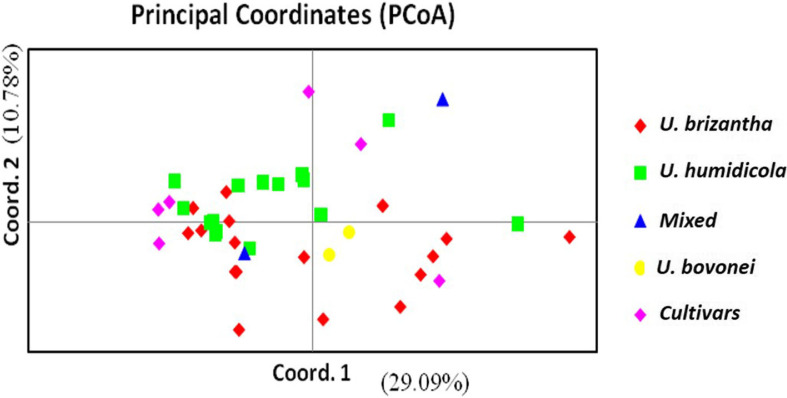
Principal coordinate analysis (PCoA) bi-plot showing the clustering of 36 *Urochloa* accessions from Tanzania and six commercial cultivars. Percentages of variation explained by the first two axes (1, 2) are 26.09 and 10.78%, respectively. (Color figure online)

**Fig. 3:**
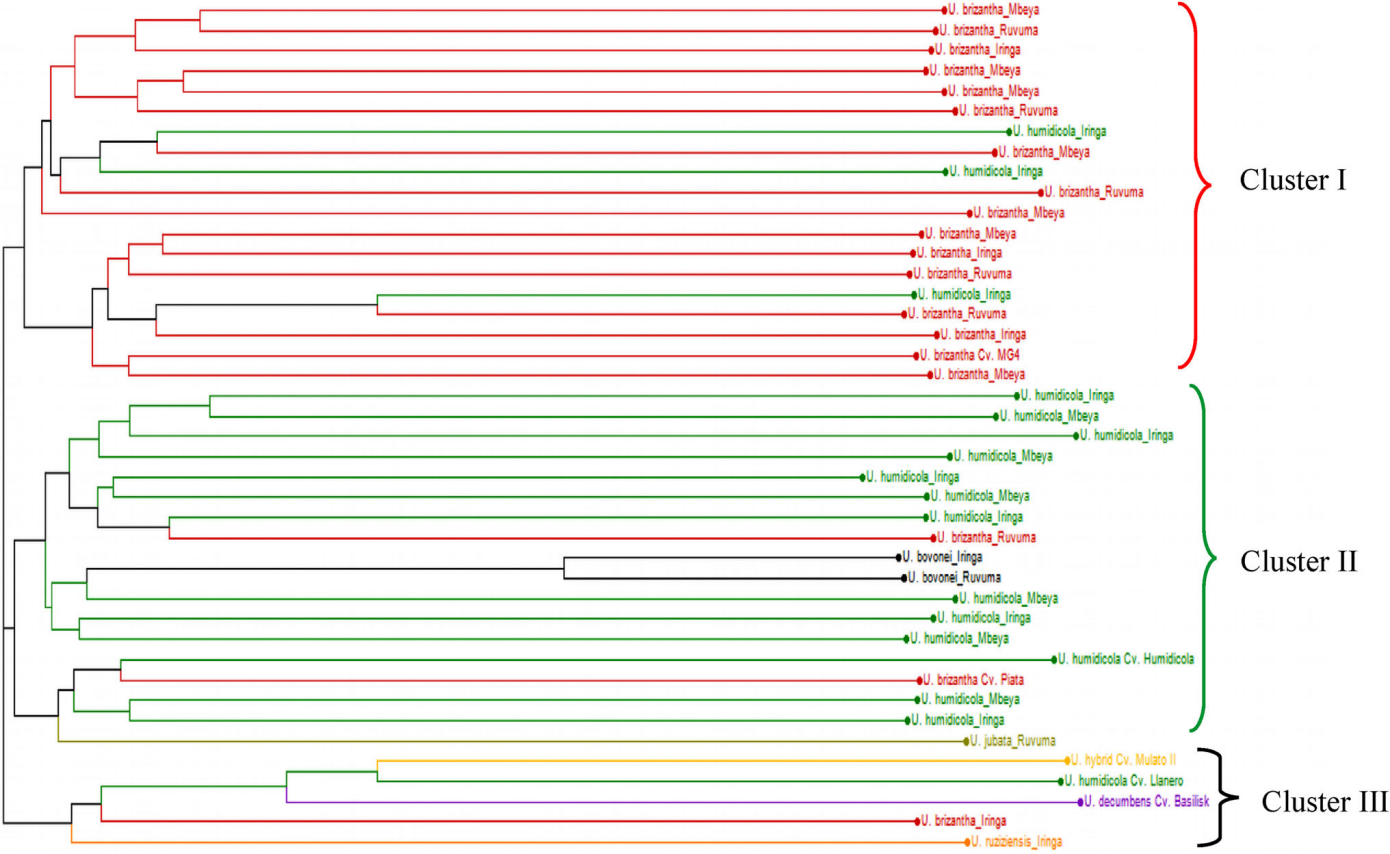
An unweighted neighbor-joining tree of 42 *Urochloa* genotypes (36 Tanzanian accessions and six commercial cultivars) using the simple matching similarity coefficient based on 24 microsatellite markers. The populations are color-coded as shown in the tree. (Color figure online)

**Fig. 4:**
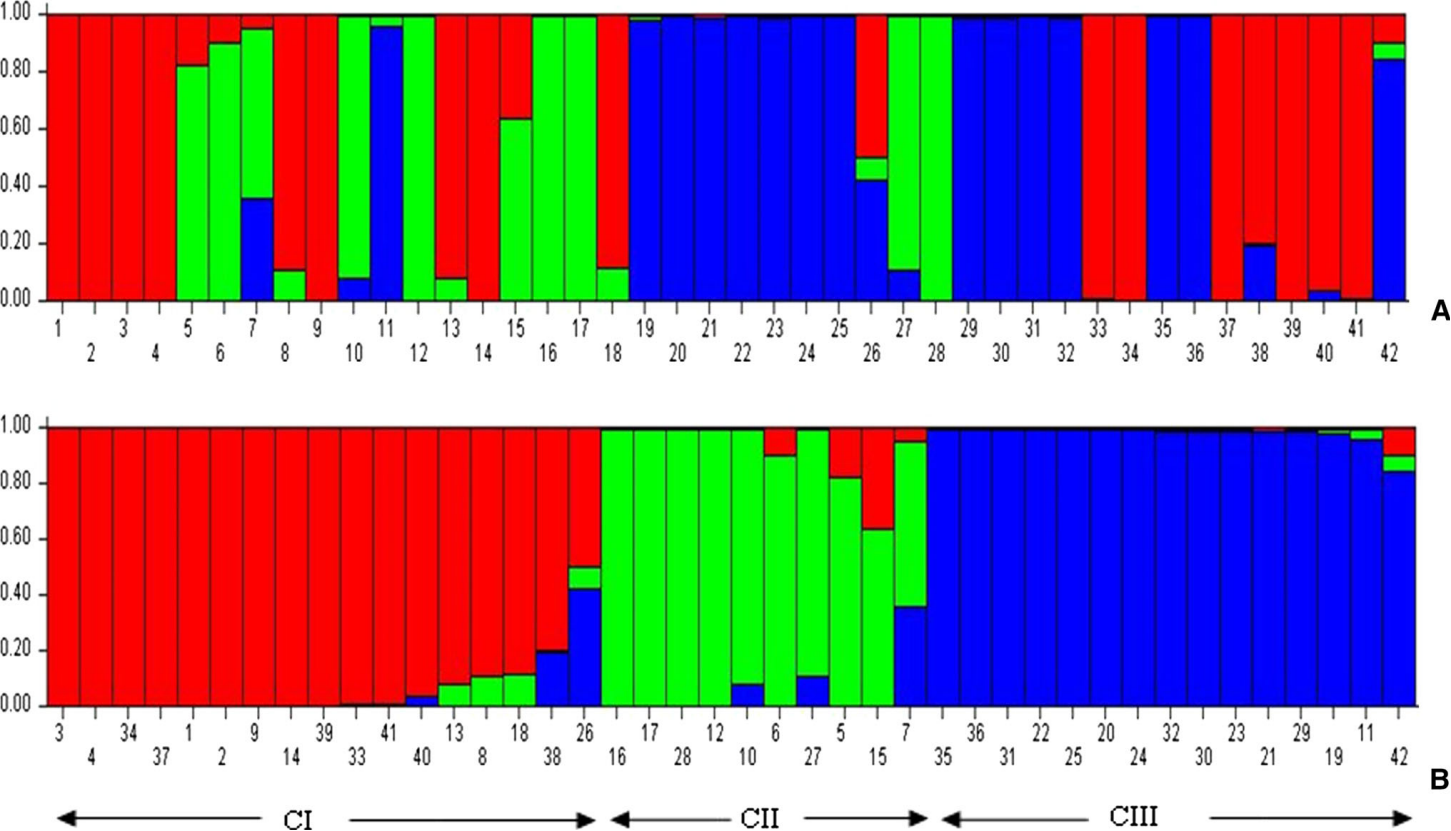
**a** Analysis performed in STRUCTURE 2.2.3 using admixture model with correlated allele frequencies. The clustering profile obtained for *K* = 3 is displayed as indicated by different colors. **b** Each of the 42 individuals is represented by a single column broken into colored segments with lengths proportional to each of the K inferred gene pools. Three major clusters of individuals were identified and are indicated by red, green and blue colors (CI = 17, CII = 10 and CIII = 15), and bars with two colors represent individuals that share allelic pools. Membership coefficients (*y*-axis) are indicated, which were used to allocate individuals into clusters. (Color figure online)

**Population structure** – The principal coordinate analysis (PCoA) bi-plot showed no distinct clustering pattern for 42 *Urochloa* genotypes studied (Fig. 2). The variations explained by axes 1 and 2 were 26.09 and 10.78%, respectively. An unweighted neighbor-joining dendrogram depicting genetic relationships among the *Urochloa* accessions and commercial cultivars showed three major clusters (Fig. 3). Of the 42 individuals including the commercial cultivars, 19, 18 and 5 individuals were grouped together in cluster I, II and III, respectively. Most of the accessions from *U. brizantha* and one commercial cultivar (MG4) grouped in cluster I, whereas most of accessions from *U. humidicola*, two accessions of *U. bovonei* and two commercial cultivars (Humidicola and Piata) grouped in cluster II. Three commercial cultivars (Basilisk, Llanero and Mulato II) and one available accession *U. ruziziensis* formed the cluster III. Overall topology of the dendrogram indicated the presence of three lineages in the *Urochloa* populations studied. A similar pattern was observed on Bayesian model-based clustering algorithm implemented in STRUCTURE software. The method of Evanno et al. (2005), implemented in STRUCTURE, predicted *K* = 3 to be the most likely number of clusters (Fig. 4).

## Discussion

Genetic diversity assessment is an essential component of any *Urochloa* breeding and conservation program. Microsatellites are among the most widely used DNA markers for many purposes such as diversity, genome mapping and variety identification (da Silva 2005). These markers have been used to study genetic diversity in different plant species (Singh et al. 2004; Joshi and Behera 2006). In this study, the extent and pattern of genetic variation among 36 Tanzanian *Urochloa* accessions were evaluated and their genetic relationships with six *Urochloa* cultivars were examined using 24 SSR markers. The SSR markers used in the study were subsets of previously published markers (Silva et al. 2013) with high polymorphic information content (PIC) values, elevated allele detection profile and proven transferability to multiple *Urochloa* species.

The average number of alleles (16.96) detected in this study was higher than that reported by Jungmann et al. (2010), Bianca et al. (2011), Silva et al. (2013) and Pessoa-Filho et al. (2015), who reported average numbers of alleles of 7.33, 4.22, 12.3 and 9 using 172 *U. brizantha*, 11 *U. ruziziensis*, 63 African Ruzigrass and 58 *U. humidicola* accessions with 15, 30, 15 and 27 SSR markers, respectively. The mean PIC value for SSR markers was high (0.79) compared to previous studies (Sousa et al. 2010; Bianca et al. 2011; Silva et al. 2013) showing high discriminating ability of these markers among tested genotypes. The detection of more alleles and high PIC values in this study could have been attributed to high diversity in Tanzanian *Urochloa* accessions, use of primers with high allele detection ability, high PIC values and proven transferability to multiple *Urochloa* species or a combination thereof. The high number of alleles detected in this study signifies high genetic variations among test *Urochloa* accessions in consistent with high genetic diversity index (0.67–0.95) (Table 3). The result is not surprising as Tanzania is within the region that represents a center of diversity for *Urochloa* species. Moreover, these 36 Tanzanian *Urochloa* accessions represent five distinct species (Table 1).

All the diversity indices are measured in this study, including the numbers of private alleles were high for *U. brizantha* population, whereas *U. bovonei* and *U. ruziziensis* populations had lower values (Table 4). As the number of different alleles and the number of private alleles depend heavily on sample size (Szpiech et al. 2008), a high number of accessions in *U. brizantha* population might have largely contributed to such results. Despite similar sample size of *U. jubata* and *U. ruziziensis*, the *U. jubata* accession had a slightly higher number of private alleles and a higher percentage of polymorphic loci, signifying that factors other than sample size also contribute to diversity indices. The observed heterozygosity was higher than expected heterozygosity for all studied *Urochloa* populations suggesting presence of many equally frequent alleles and the high genetic variability in the populations indicating high value of these genetic resources in *Urochloa* improvement and conservation program. Mixing of two previously isolated *Urochloa* populations could be another possibility for higher observed heterozygosity than expected.

Genetic distance is the measure of the allelic substitutions per locus that have occurred during the separate evolution of two populations or species (Woldesenbet et al. 2015). The Nei unbiased genetic distance between *U. brizantha* and *U. humidicola* was smaller, while larger genetic distance was observed between *U. bovonei* and the commercial cultivars. The genetic closeness of two populations could be due to interspecific hybridization that has occurred throughout their evolution, which favors allele sharing (Cidade et al. 2013). The large genetic distance observed between *U. bovonei* and commercial cultivars could be attributed by lack of genetic similarity as five commercial cultivars used in this species are from three species (*U. brizantha*, U. decumbens Stapf and *U. humidicola*), while commercial cultivar, Mulato II, is a product of three-way cross of *U. brizantha*, U. decumbens and *U. ruziziensis*. Two species, i.e., *U. jubata* and *U. ruziziensis*, were not included in this analysis due to insufficient sample size.

The AMOVA test showed major and significant (92%; *P* = 0.001) contribution of within-individual difference to a total variation, whereas among-individual and among-population differences contributed 3 and 5%, respectively. The high level of genetic variation within species observed in our study was similar to that reported for Ruzigrass (Pessoa-Filho et al. 2015). These results are also in agreement with other studies (Bianca et al. 2011; Garcia et al. 2013; Teixeira et al. 2014). The high level of genetic variation within individual in a population could be attributed to genetic drift, mutation and environment conditions (Young et al. 2000). As the *Urochloa* population/species in this study are composed of genotypes originating from different locations with different geographical and environment conditions, a high within-population difference was expected. There was a low genetic variation among *Urochloa* accessions in consistent with the high genetic indices as evidenced by relatively low fixation index (*F*_ST_ = 0.05) among populations and high number of migration (Nm = 4.7) per generation (Slatkin 1981; Caccone 1985; Walples 1987). A low genetic differentiation among *Urochloa* populations was anticipated because of apomictic mode of reproduction, polyploidy-triggered meiotic anomalies obstructing sexual reproduction and dispersion of plant propagules by migratory herbivores and birds. Of five *Urochloa* species analyzed in this study, four (*U. brizantha*, *U. humidicola*, *U. bovonei* and *U. jubata*) are polyploid (Boldrini et al. 2009; Bianca et al. 2011) and *U. ruziziensis* is diploid with sexual mode of reproduction (Pessoa-Filho et al. 2015). Polyploid plants can effectively colonize and occupy different habitats favoring no genetic differentiation among *Urochloa* populations (De Wet 1980). This has also been observed in other apomictic polyploid forages such as Paspalum notatum Fluegge (Cidade et al. 2013).

In PCoA, no distinct clusters were observed; however, STRUCTURE and the unweighted neighbor-joining algorithm analyses consistently revealed three major clusters (Figs. 3, 4). Cluster I was mainly composed of *U. brizantha* accessions (15 out of 17), while most *U. humidicola* accessions (12 out of 15) were found in cluster II and 3 of 6 commercial cultivars were found in cluster III. Two accessions of *U. bovonei* and one of *U. jubata* were found in cluster II, but in different sub clusters. Although *U. ruziziensis* was found in cluster III, it is a bit far from the rest of accessions (Fig. 3). This is as expected because it is only one accession included in this study with diploid genome and sexual mode of reproduction. The accessions included in the study grouped together irrespective of their geographical origin indicating accessions from different geographical regions share the allelic pool (Sousa et al. 2010). However, a little admixture of accessions from different allelic pools was observed in all clusters showing possible interspecific hybridization that might have occurred during the evolution favoring allele sharing, or could be due to the error while assigning species. This study revealed a high genetic diversity in Tanzanian *Urochloa* accessions compared to six commercial *Urochloa* cultivars. The SSR markers used in this study were highly informative to assess genetic diversity in *Urochloa* species. The *Urochloa* accessions did not cluster according to the geographical regions but clustered by their genetic background. The accessions belonging to *U. brizantha* were more diverse than those from other four species and commercial cultivars, which can be tapped and used in conservation and breeding programs, especially in developing improved *Urochloa* varieties and hybrids that can produce high biomass and withstand well to biotic and abiotic environmental conditions. The cultivars and sexual diploid *U. ruziziensis* from cluster III can be used in future crosses with other accessions from cluster I and II depending on their ploidy to obtain heterosis in the progeny. As the *Urochloa* accessions analyzed in this study represent only 3 of 31 regions of Tanzania, collecting *Urochloa* germplasm from a wider geographical area is necessary to catalog the genetic variation of *Urochloa* in the country.

## Data accessibility

Data are available from the Dryad Digital Depository (Kuwi, Kyalo, Mutai, Mwilawa, Hanson, Djikeng and Ghimire, 2018): DOI: https://doi.org/10.5061/dryad.42075m1.
